# Early manipulation under anaesthesia for stiffness following total knee arthroplasty is associated with a greater gain in knee flexion

**DOI:** 10.1007/s00167-022-07128-7

**Published:** 2022-08-30

**Authors:** Richard Rahardja, Aziz Mehmood, Brendan Coleman, Jacob T. Munro, Simon W. Young

**Affiliations:** 1 Department of Surgery, Faculty of Medical and Health Sciences, University of Auckland, Auckland, New Zealand; 2 Department of Orthopaedic Surgery, Middlemore Hospital, Auckland, New Zealand; 3 Department of Orthopaedic Surgery, Auckland City Hospital, Auckland, New Zealand; 4 Department of Orthopaedic Surgery, North Shore Hospital, Auckland, New Zealand

**Keywords:** Total knee arthroplasty, TKA, Manipulation under anaesthesia, MUA, Stiffness

## Abstract

**Purpose:**

This study aimed to identify the risk factors for manipulation under anaesthesia (MUA) following total knee arthroplasty (TKA) and whether performing an ‘early’ MUA within 3 months leads to a greater improvement in range of motion.

**Methods:**

Primary TKAs performed between 2013 and 2018 at three tertiary New Zealand hospitals were reviewed with a minimum follow-up of 1 year. Clinical details of patients who underwent MUA were reviewed to identify the knee flexion angle prior to and following MUA. Multivariate analysis identified the risk factors for undergoing MUA and compared flexion angles between ‘early’ (< 3 months) and ‘late’ MUA (> 3 months).

**Results:**

A total of 7386 primary TKAs were analysed in which 131 underwent an MUA (1.8%). Patients aged < 65 years were two times more likely to undergo MUA compared to patients aged ≥ 65 years (2.5 versus 1.3%, *p* < 0.001; adjusted HR = 2.1, *p* < 0.001). There was no difference in the final flexion angle post-MUA between early and late MUA (104.7° versus 104.1°, *p* = 0.819). However, patients who underwent early MUA had poorer pre-MUA flexion (72.3° versus 79.6°, *p* = 0.012), and subsequently had a greater overall gain in flexion compared to those who underwent late MUA (mean gain 33.1° versus 24.3°, *p* < 0.001).

**Conclusion:**

Younger age was the only patient risk factor for MUA. Patients who underwent early MUA had similar post-MUA flexion, but had poorer pre-MUA flexion compared to those who underwent late MUA. Subsequently, a greater overall gain in flexion was achieved in those who underwent early MUA.

**Level of evidence:**

III.

## Introduction

Total knee arthroplasty (TKA) is effective in providing pain relief and restoring function to the arthritic knee [18]. Despite advances in surgical technique, implant design and rehabilitation, complications such as arthrofibrosis and stiffness may affect more than 20% of patients [9, 21, 22]. Patient factors including age, sex and ethnicity are commonly associated with a higher prevalence of stiffness, but there remains conflicting evidence on the effect of obesity and comorbidities [2, 5, 6, 10, 17, 20, 22].

Initial management of stiffness following TKA involves physiotherapy and rehabilitation exercises to regain motion. However, if stiffness persists in the absence of infection or component malposition, then a reoperation may be necessary. This includes either a manipulation under anaesthesia (MUA), arthroscopic lysis of adhesions, open lysis of adhesions or revision TKA. MUA is considered the first-line surgical treatment for stiffness as it is the least invasive procedure and may achieve equivalent results to arthroscopic lysis [3, 7]. However, there is significant debate on the optimal timing of MUA [9, 22]. Most clinicians argue that the ideal time for performing an MUA is within 3 months of the primary TKA [3, 9, 15, 22]. Though other clinicians have argued for MUA to be performed as early as within 2 weeks [4], or as late as 6 months after the primary TKA [11, 22].

This study aimed to clarify the risk factors for undergoing MUA following primary TKA and investigate whether performing an MUA within 3 months of primary TKA results in a significantly greater gain in range of motion (ROM). It was hypothesised that patients who undergo early MUA within 3 months would achieve a greater gain in flexion compared to patients who undergo late MUA more than 3 months after primary TKA.

## Materials and methods

Ethical approval was obtained from the Auckland Health Research Ethics Committee (AHREC) and locality approval obtained from each tertiary hospital prior to data collection. This study received exemption from the Health and Disability Ethics Committee (HDEC) review as an audit activity.

### Study design and setting

This study was a multicentre, retrospective review of patients who underwent TKA at three tertiary referral hospitals in Auckland, New Zealand. The study period was 1st January 2013 to 31st December 2018, allowing for a minimum follow-up of 1 year. There were 7412 TKAs performed during this period, of which 7386 were included in the study. Patients were excluded if they had deceased within 12 months of the TKA (26 knees).

### Manipulation under anaesthesia and flexion angles

Patients who had undergone an MUA were identified through a coding search using the International Classification of Diseases (ICD-10) discharge coding at each tertiary hospital. For every MUA identified, a manual review of patient electronic notes was performed, including any admission summaries, operation notes, discharge summaries or follow-up letters. This allowed the authors to retrieve the knee flexion angle documented by the attending surgeon prior to the MUA, directly after the MUA, and goniometer recordings at subsequent clinic follow-up. Timing of MUA was classified as being early (< 3 months from TKA) or late (> 3 months from TKA) [15].

### Predictor variables

Patient demographic and intraoperative surgical data were accessed and retrieved from the New Zealand Joint Registry (NZJR). The patient factors of interest in this study included age, sex, American Society of Anaesthesiologists (ASA) score, body mass index (BMI), a history of cancer and the hospital that the primary TKA was performed in. The surgical factors of interest included the use of computer-assisted navigation during the primary TKA, surgical duration and whether the TKA was performed by a consultant or trainee surgeon.

### Statistical analysis

Descriptive statistics were provided as mean values with standard deviation (SD) or median values with interquartile ranges (IQR). Continuous variables were assessed for normality through visualisation of Q–Q plots and histograms. Univariate analysis was performed via Chi-square test for categorical variables and Student *t*-test or Mann–Whitney *U* test for continuous variables. Multivariate Cox proportional hazards regression was performed to compute hazard ratios (HR) with 95% confidence intervals (CI) to evaluate independent predictors for MUA. Multivariate linear regression was performed to compare the mean flexion angles pre- and post-MUA as well as the mean gain in flexion in degrees. Results were considered statistically significant at *p* < 0.05. All analyses were performed using IBM SPSS Statistics version 25.

## Results

A total of 7386 primary TKAs were analysed in which 131 patients underwent subsequent MUA (1.8%). The median time to MUA was 78 (52–121) days. The earliest MUA was performed at 5 days post-TKA and the latest MUA was performed at 1460 days post-TKA. Patient demographics are shown in Table 1.

**Table 1:** Baseline demographics and univariate analysis of patients undergoing MUA

	Total		Patients undergoing MUA		
Demographic	*n*	%	*n*	%	*p* value
Eligible patients	7386		131	1.77	
Sex					0.75
Male	3202	43.4	55	1.72	
Female	4184	56.6	76	1.82	
Hospital					< 0.001
Hospital A	2316	31.4	22	0.95	
Hospital B	1613	21.8	18	1.12	
Hospital C	3457	46.8	91	2.63	
Age (years)					
Continuous					
Mean ± SD	68.3 (± 9.4)		65.6 (± 9.3)		< 0.001
Categorical					
< 65	2843	38.5	72	2.53	< 0.001
≥ 65	4543	61.5	59	1.30	
ASA score					0.482
1 or 2	4924	66.7	91	1.85	
3 or 4	2411	32.6	39	1.62	
NR	51	0.7	1	1.96	
BMI					
Continuous					
Mean ± SD	32.3 (± 6.4)		32.0 (± 5.5)		0.748
Categorical					
< 25	634	8.6	7	1.10	0.33
25–29.9	1989	26.9	38	1.91	
30–34.9	2002	27.1	41	2.05	
≥ 35	2390	32.4	37	1.55	
NR	371	5.0	8	2.16	
Computer-assisted navigation					< 0.001
Yes	3379	45.7	83	2.46	
No	4007	54.3	48	1.20	
Surgical duration (min)					
Continuous					
Median (IQR)	87 (70–107)		85 (69–105)		0.287
Categorical					
≤ 100	4847	65.6	90	1.86	0.487
> 100	2338	31.7	38	1.63	
NR	201	2.7	3	1.49	
Surgeon level					0.42
Consultant	5763	78.0	106	1.84	
Trainee	1623	22.0	25	1.54	
Cancer					0.192
Yes	761	10.3	18	2.37	
No	6625	89.7	113	1.71	

On multivariate analysis (Table 2), the risk of MUA was over two times higher in patients younger than 65 years (adjusted HR = 2.11, *p* < 0.001) and in primary TKAs performed in Hospital C (adjusted HR = 2.47, *p* = 0.002).

**Table 2:** Multivariate analysis: predictors of MUA

Factor	HR (95% CI)	*p* value
Sex		
Male	0.78 (0.54–1.13)	0.192
Female	Reference	
Age		
< 65	2.11 (1.45–3.07)	< 0.001
≥ 65	Reference	
Hospital		
Hospital A	Reference	0.86
Hospital B	1.06 (0.54–2.11)	
Hospital C	2.47 (1.41–4.34)	0.002
ASA score		
1 or 2	0.96 (0.64–1.44)	0.846
3 or 4	Reference	
BMI		
< 25	Reference	
25–29.9	1.76 (0.78–3.94)	0.172
30–34.9	1.70 (0.76–3.81)	0.197
≥ 35	1.18 (0.52–2.70)	0.690
Computer-assisted navigation		
Yes	1.46 (0.95–2.27)	0.088
No	Reference	

In the 131 patients who underwent an MUA, the knee flexion angle documented immediately before and after the MUA was analysed to calculate the overall gain in flexion (Table 3). Interestingly, patients who underwent an early MUA had significantly poorer pre-MUA flexion compared to patients who underwent late MUA (72.3° versus 79.6°, *p* = 0.012). Although there was no difference in the final flexion angle post-MUA (104.7° versus 104.1°, *p* = 0.819), a greater overall gain in flexion was observed in patients who underwent early MUA (mean gain 33.1° versus 24.3°, *p* < 0.001). After adjusting for patient age, sex and the surgeon level, performing a late MUA was associated with a smaller overall gain in knee flexion when compared to performing an early MUA (mean adjusted difference = − 7.8, *p* = 0.005, Table 4).

**Table 3:** Range of motion pre-, post-MUA and gain in flexion

	Total	Pre-MUA		Post-MUA		Gain in flexion	
Demographic	*n*	Mean flexion	*p* value	Mean flexion	*p *value	Mean gain	*p *value
Eligible patients	131	75.5 ± 16.2		104. 4 ± 13.8		29.1 ± 15.2	
Timing of MUA							
Early ≤ 3 months	72	72.3 ± 16.6	0.012	104.7 ± 13.5	0.819	33.1 ± 16.2	< 0.001
Late > 3 months	59	79.6 ± 14.9		104.1 ± 14.2		24.3 ± 12.5	
Sex							
Male	55	78.3 ± 17.2	0.119	104.6 ± 14.8	0.894	27.4 ± 15.3	0.318
Female	76	73.7 ± 15.3		104.3 ± 13.1		30.28 ± 15.2	
Hospital							
Hospital A	22	69.2 ± 18.1	0.182	96.9 ± 12.3	0.003	25.7 ± 13.1	0.507
Hospital B	18	79.6 ± 16.7		107.5 ± 14.2		27.9 ± 14.2	
Hospital C	91	76.7 ± 15.7		106.7 ± 12.3		30.0 ± 15.8	
Age (years)							
Categorical							
< 65	72	76.6 ± 15.4	0.446	103.7 ± 12.8	0.529	26.9 ± 13.7	0.076
≥ 65	59	74.3 ± 17.2		105.3 ± 15.1		31.2 ± 16.7	
ASA score							
1 or 2	91	75.3 ± 16.8	0.911	104.8 ± 12.7	0.605	29.4 ± 15.5	0.776
3 or 4	39	75.7 ± 14.9		103.3 ± 16.6		28.5 ± 15.0	
BMI							
< 25	7	82.5 ± 8.8	0.117	105.8 ± 18.8	0.331	21 ± 16.0	0.29
25–29.9	38	77.0 ± 16.2		107.7 ± 12.8		30.8 ± 14.9	
30–34.9	41	78.7 ± 17.5		105.4 ± 13.3		26.1 ± 13.2	
≥ 35	37	71.1 ± 13.3		102.2 ± 12.1		30.7 ± 16.3	
Computer-assisted navigation							
Yes	83	78.8 ± 15.8	0.252	107.8 ± 11.8	< 0.001	30.8 ± 15.4	0.089
No	48	73.3 ± 16.8		98.3 ± 15.0		25.8 ± 14.5	
Surgical duration (min)							
Categorical							
≤ 100	90	75.0 ± 17.6	0.453	104.5 ± 14.0	0.65	29.6 ± 16.0	0.801
> 100	38	77.4 ± 12.6		105.7 ± 12.7		28.9 ± 13.7	
Surgeon level							
Consultant	106	74.7 ± 16.6	0.116	105.0 ± 12.9	0.272	30.6 ± 15.2	0.02
Trainee	25	80.2 ± 13.7		101.5 ± 17.5		22.3 ± 13.6	
Cancer							
Yes	18	79.7 ± 11.6	0.275	110.2 ± 13.4	0.061	28.9 ± 17.0	0.968
No	113	74.9 ± 16.7		103.5 ± 13.7		29.1 ± 15.0

**Table 4:** Multivariate analysis: gain in flexion

Factor	Correlation coefficient	95% confidence interval	*p* value
Sex			
Male	Reference		
Female	2.7	− 2.7 to 8.1	0.32
Age			
< 65	Reference		
≥ 65	3.8	− 1.5 to 9.1	0.159
Consultant			
Yes	Reference		
No	− 5.8	− 12.7 to 1.1	0.099
Timing of MUA			
Early ≤ 3 months	Reference		
Late > 3 months	− 7.8	− 13.2 to − 2.4	0.005

## Discussion

The most important finding of this study was that patients who underwent early MUA had poorer knee flexion prior to undergoing MUA and subsequently achieved a greater overall gain in knee flexion compared to patients who underwent late MUA.

Stiffness following primary TKA is a common complication that can significantly impair the ability for patients to perform daily activities [1]. In this study, the mean pre-MUA knee flexion angle was 75.5° with 89% (*n* = 116) of patients having a pre-MUA flexion of less than 90°. This indicates that most patients would have struggled to perform basic tasks such as climbing stairs, rising from a chair and walking which require knee flexion of between 67 and 93° [8, 12, 13, 19]. However, post-operative stiffness is difficult to define. Most studies analyse arthrofibrosis and stiffness as either loss of terminal extension, flexion less than 90°, poorer range of motion when compared to before the primary TKA or the need to undergo MUA or reoperation [9, 12, 19, 21]. Younger age is the most commonly reported independent predictor of stiffness following primary TKA [10, 12, 16, 17, 20, 22]. In this study, younger age was the only patient risk factor for undergoing an MUA, with two times higher risk of MUA observed in patients younger than 65 years. Younger patients may have greater expectations and functional demands which may make them more likely to undergo MUA to achieve greater ROM. In contrast, the literature has reported conflicting results on the association between BMI and stiffness. In a study of 391 primary TKA with 65 MUAs, Gadinsky et al. performed univariate analysis and suggested a non-linear increase in the rate of MUA with higher BMI (10% for a BMI < 25 kg/m^2^, 19% for a BMI of 25–29.9 kg/m^2^, 20% for a BMI of 30–34.9 kg/m^2^, and 15% for a BMI of ≥ 35 kg/m^2^) [5]. However, in a multivariate analysis using the same BMI cutoffs, this study found that BMI did not independently predict the risk of MUA. A multivariate analysis performed in 3182 TKAs by Issa et al. and a study of 1729 TKAs by Newman et al. similarly found no association [10, 16].

Although the optimal timing of MUA is widely debated between surgeons, the general consensus is that it does influence the outcome of MUA [3, 22]. An international consensus provided by the Knee Joint Fibrosis Working Group suggested that performing an MUA between 3 and 6 months is the optimal time period as this when maturation of adhesive tissue occurs [11, 14]. Performing an MUA beyond 6 months may not be sufficient to break the fibrosis [11]. This study analysed the importance of MUA timing using a cutoff of 3 months to categorise MUAs into ‘early’ or ‘late’, therefore, allowing for comparison to other studies in the literature. Although there was no difference in final flexion post-MUA between the two cohorts, patients in the ‘early’ MUA cohort had poorer flexion angles pre-MUA and ultimately had a greater gain in overall flexion compared to patients in the ‘late’ cohort. These findings are identical to that of Namba et al. who analysed 195 MUAs in 9640 primary TKAs [15]. Using the same cutoff of 3 months, they found that final flexion post-MUA was similar (101.4° vs 98.0°), but patients in the ‘early’ cohort had poorer flexion pre-MUA (68.4° vs 81.0°, *p* < 0.001) and a subsequently greater overall gain in flexion (mean gain = 31.6° vs 19.5°). Using a cutoff of 6 weeks, Newman et al. analysed 62 MUAs performed in 1729 primary TKAs and comparably found no difference in final flexion post-MUA (115.3° vs 115.3°), but observed poorer pre-MUA knee flexion in the ‘early’ MUA cohort (59.4° vs 73.5°, *p* = 0.019) [16]. It is unclear why patients undergoing ‘early’ MUA are associated with poorer pre-MUA flexion angles; however, it may be related to surgeon preference and hospital resources. In patients who have significantly impaired knee flexion, surgeons may opt to be more aggressive in their management of the stiff knee and proceed to an ‘early’ MUA to salvage outcomes. Patients with a less severe limitation of flexion may be given more time to improve with physical therapy and only proceed to MUA if a lack of improvement is seen at subsequent follow-up. The influence of surgeon preference on MUA timing may be demonstrated by the finding that patients who had their primary TKA performed in Hospital C were more likely to undergo an MUA when compared to primary TKAs performed in Hospital A.

### Limitations

This study is limited to analysing the rate of MUA as a proxy measure for stiffness or arthrofibrosis. As not all patients who suffer from arthrofibrosis will proceed to a MUA, this will underestimate the true prevalence of stiffness. The decision by both the patient and surgeon to proceed to a MUA may depend on multiple factors, such as pre-operative range of motion, patient comorbidities, and functional demands. However, MUA represents a well-defined outcome measure following TKA that involves significant healthcare resources, and analysis of this outcome allows for comparison to other studies. It is also easily and accurately recorded. Second, this study was a retrospective analysis and was unable to randomly allocate patients to undergo early or late MUA. However, a strength of the present study is the large number of primary TKAs and MUAs, allowing for greater statistical power and a patient population that may be difficult and expensive to obtain in a randomised controlled trial. Lastly, although this study analysed the impact of MUA timing on the knee flexion angles prior to and after MUA, it was unable to analyse any loss of knee flexion that may occur over time after MUA. Further studies are required to clarify the association between time since MUA and knee flexion.

The clinical relevance of this study is that performing early MUA for stiffness following primary TKA may achieve a greater gain in knee flexion compared to late MUA.

## Conclusion

Younger age was the only patient factor that independently predicted the risk of MUA. Patients undergoing an early MUA within 3 months had similar post-MUA flexion, but had poorer pre-MUA flexion compared to patients undergoing late MUA. Subsequently, a greater overall gain in flexion was observed in patients who underwent early MUA.
